# Targeting TMEM16A/ANO1 inhibits the progression of BRAF mutant (V600E) melanoma through the MEK/ERK and AKT signaling pathways

**DOI:** 10.1016/j.gendis.2023.101153

**Published:** 2023-10-27

**Authors:** Meiyun Shi, Jiahui Wang, Xingyu Huang, Linsu Liu, Lutao Liu, Na Zhou, Xinqi Shan, Huaqun Chen

**Affiliations:** Jiangsu Key Laboratory for Molecular and Medical Biotechnology, College of Life Sciences, Nanjing Normal University, Nanjing, Jiangsu 210023, China

The BRAF (V600E) mutation is common in malignant melanoma patients and drives constitutive activation of the MEK/ERK signaling pathway and cancer progression.^1^ To date, precision therapies targeting the BRAF/MEK/ERK pathway, including BRAF inhibitor (BRAFi) monotherapy using vemurafenib (VMF) and dabrafenib and combination therapy using dabrafenib and the MEKi trametinib, have been developed and have led to great advances in malignant melanoma therapy.^2^ Unfortunately, many tumors initially responsive to these drugs develop resistance, which leads to patient death. Therefore, the exploration of alternative targets is important and urgently needed to improve the treatment of malignant melanoma patients. Studies have shown that abnormally high expression levels of TMEM16A (also called ANO1), a calcium-activated chloride channel (CaCC), contribute to the occurrence and proliferation of many kinds of tumor cells.^3^ Here, we report that TMEM16A/ANO1 is significantly up-regulated in A375 human malignant melanoma cells, which carry the BRAF(V600E) mutation. *In vitro*, inhibition of TMEM16A by two CaCC inhibitors (T16Ainh-A01 and CaCCinh-A01) and shRNA-mediated knockdown of TMEM16A suppressed the proliferation of A375 cells. *In vivo*, the growth of engrafted A375 tumors in nude mice was mitigated by TMEM16A knockdown. Mechanistically, the activation of the MEK/ERK and AKT signaling pathways was inhibited.

We first examined available databases for assessing the up-regulation of TMEM16A at the protein and mRNA levels in melanoma tissues and assessed the potential correlation between the overall survival of the malignant melanoma patients and the TMEM16A expression levels. Analysis of the Human Protein Atlas (http://www.proteinatlas.org) showed that TMEM16A protein expression was not detected in melanocytes, but weak signals were detected in melanoma samples of some patients (Fig. 1A). The data provided by UALCAN (https://ualcan.path.uab.edu) revealed that the mRNA expression levels of the TMEM16A gene were increased in primary (*n* = 104) and metastatic melanoma tissues (*n* = 368) compared with normal tissues (*n* = 1) (Fig. 1B). Survival analysis showed that the survival probability at up to three years after diagnosis was obviously decreased in patients with high mRNA expression levels (*n* = 81) in melanoma tissues compared with those with low mRNA expression levels (*n* = 21) (http://www.proteinatlas.org) (*P* = 0.021) (Fig. 1C). Another database (gepia.cancer-pku.cn) also showed that the overall survival rate of malignant melanoma patients tended to be lower in the group with high TMEM16A expression (*n* = 229) than in the group with low TMEM16A expression (*n* = 229). These results suggest that high TMEM16A expression may be correlated with the progression of malignant melanoma.

**Figure 1:**
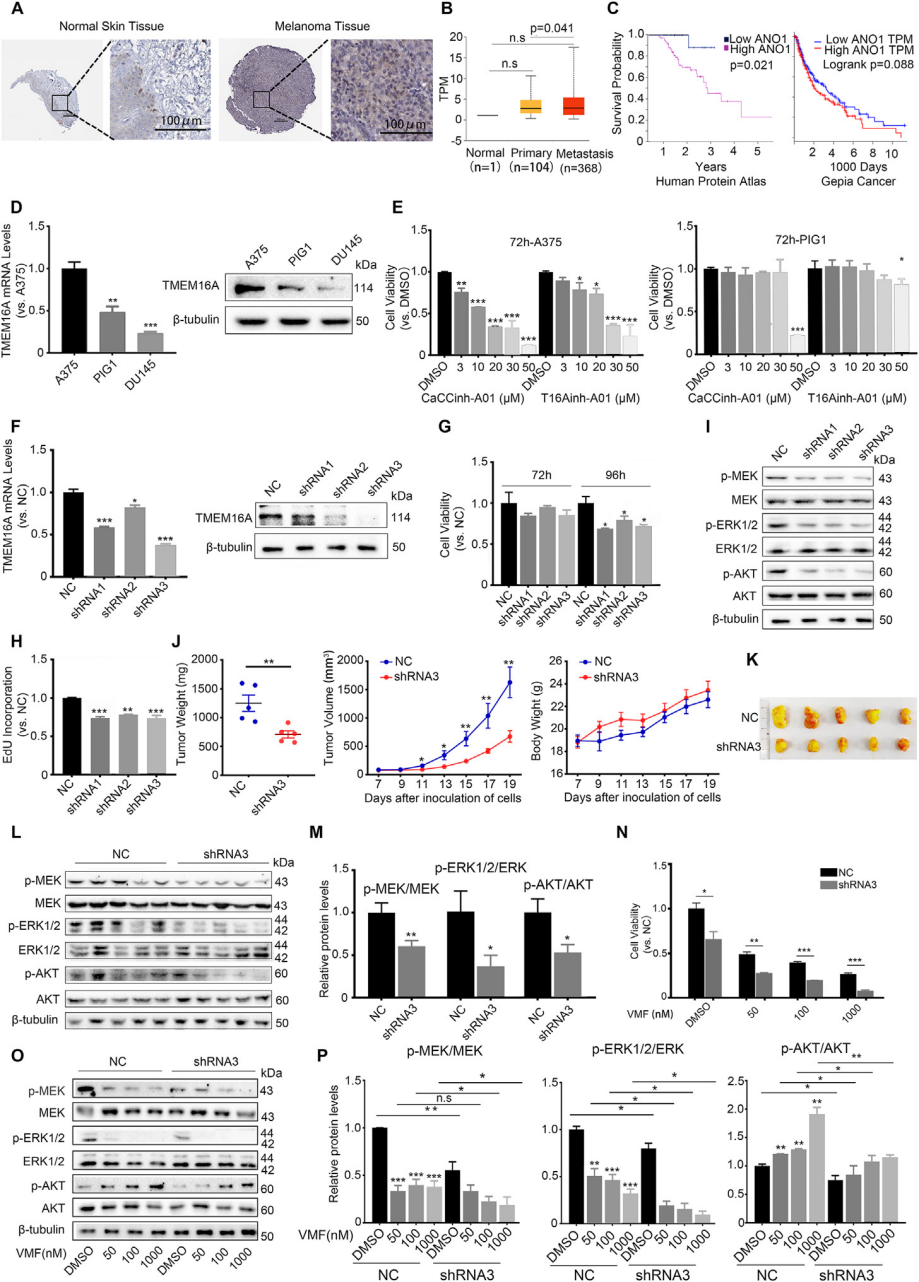
Inhibition of TMEM16A/ANO1 suppresses melanoma progression through decreased MEK-ERK and AKT activation. **(A)** Immunohistochemical staining of TMEM16A protein in normal and melanoma tissues. **(B)** TPM values for the TMEM16A gene in normal tissues and melanoma tissues. **(C)** Overall survival probability curve for melanoma patients with low or high expression of TMEM16A. **(D)** Up-regulated expression of TMEM16A protein in A375 cells. **(E)** CaCCinh-A01 or T16Ainh-A01 inhibited the growth of A375 cells, as determined by MTT assay. **(F)** Decreased expression of TMEM16A in TMEM16A shRNA lentivirus-transduced A375 cells. **(G)** Growth inhibition in A375 melanoma cells infected with TMEM16A shRNA lentiviruses. **(H)** EdU assay for A375 cells infected with shRNA lentiviruses. **(I)** Western blotting analysis of phosphorylated MEK/ERK1/2 and AKT in A375 cells transduced with shRNA lentiviruses. **(J)** Xenograft tumor growth in nude mice and body weight of the mice with xenografted tumors. The tumor data are shown as the mean volume or weight ± standard error of the mean (mm^3^ or mg). *n* = 5. ^∗^*P* < 0.05, ^∗∗^*P* < 0.01. **(K)** Comparisons of tumors that were removed on the 19th day from nude mice. **(L, M)** Western blotting analysis of phosphorylated MEK/ERK1/2 and AKT in xenograft tumors with (shRNA3) or without knockdown of TMEM16A (NC). *n* = 5. ^∗^*P* < 0.05, ^∗∗^*P* < 0.01. **(N)** An additive suppressing effect of TMEM16A knockdown on the proliferation of A375 cells treated with indicated concentrations of VMF for 96 h, as determined by MTT assay. **(O, P)** Western blotting analysis of phosphorylated MEK/ERK1/2 and AKT in TMEM16A knockdown A375 cells treated with indicated concentrations of VMF for 1 h, *n* = 3 for all the *in vitro* experiments. ^∗^*P* < 0.05, ^∗∗^*P* < 0.01, ^∗∗∗^*P* < 0.001.

We next assessed differential TMEM16A expression in A375 cells and normal cells. The results showed marked increases in TMEM16A protein and mRNA levels in A375 cells compared with the corresponding normal control nevus cell line PIG1 as well as another cancer cell line (DU145) reported previously^4^ (Fig. 1D). To determine the functions of TMEM16A in A375 cells, we assessed the effects of two CaCC inhibitors on cell proliferation. MTT assay data showed that the inhibition of TMEM16A by either inhibitor induced a significant reduction in the proliferation of A375 cells compared with the DMSO control. In contrast, no apparent effects of the inhibitors on the proliferation of PIG1 cells were observed, except for CaCCinh-A01 at a relatively high concentration (50 μM) (Fig. 1E).

To further investigate the role of TMEM16A in the proliferation of A375 cells, three TMEM16A shRNA oligos (shRNA1, shRNA2, and shRNA3) were designed and expressed *via* lentiviral transduction to knock down the expression of TMEM16A. The decrease in TMEM16A expression after shRNA lentivirus transduction was demonstrated by qRT-PCR and western blotting (Fig. 1F). Accordingly, obvious inhibition of cell proliferation was observed in A375 cells at 96 h after infection with the lentiviruses (Fig. 1G). The suppressed proliferation of TMEM16A knockdown A375 cells was further validated by an EdU incorporation assay (Fig. 1H; Fig. S1). Taken together, the above findings indicated that the proliferation of A375 cells was associated with up-regulation of TMEM16A.

To explore the mechanisms underlying the inhibition of melanoma cell proliferation by TMEM16A knockdown, we examined the downstream signaling pathways contributing to cancer growth induced by TMEM16A overexpression, such as the MEK/ERK and AKT pathways.^3^ As shown in Figure 1I, the knockdown of TMEM16A apparently suppressed the activation of MEK/ERK and AKT in A375 cells infected with the TMEM16A shRNA lentiviruses compared with those transfected with the NC control.

To examine the effect of TMEM16A on malignant melanoma growth *in vivo*, we infected A375 cells with TMEM16A shRNA3 lentiviruses to knock down TMEM16A expression in the cells. Animals were divided into two groups for *sc* injection of TMEM16A shRNA3- (shRNA3) or NC shRNA (NC)-transduced A375 cells. Tumor volume, final tumor weight, and body weight were recorded every other day until the 19th day after the injection of the cells when the mice were sacrificed. As shown in Fig. 1J and K, shRNA3-transduced A375 cells developed into xenograft tumors with a smaller average volume than NC tumors. The average weight of tumors in the shRNA3 group was greatly decreased compared with that in the NC group. No apparent difference was detected in the body weight of the two groups. These data demonstrated that a decrease in TMEM16A expression significantly suppressed the growth of xenograft tumors *in vivo*. The NC tumors displayed relatively high levels of constitutive phosphorylation of MEK/ERK and AKT, whereas the phosphorylation levels were significantly lower in the tumors of the shRNA3 group (Fig. 1L, M). These results indicate a reduction in the activated MEK/ERK and AKT levels in tumors with TMEM16A knockdown.

To validate the possibility that TMEM16A is a potent alternative target for the treatment of malignant melanoma, we evaluated the effects of TMEM16A knockdown on the inhibitory growth effect of VMF on A375 cells. The results showed that the proliferation of A375 cells was significantly suppressed by VMF, consistent with the previous study (Fig. S2). Importantly, when shRNA3 was combined with VMF, an additive inhibitory effect on the proliferation of A375 cells was observed (Fig. 1N). As reported previously, VMF reduced the phosphorylation levels of MEK and ERK in A375 cells but elevated the phosphorylation level of AKT. TMEM16A shRNA3 introduction induced an additional decline in MEK and ERK activation levels. Strikingly, it also significantly reduced the AKT activation induced by VMF (Fig. 1O, P). Given that induced AKT activation is associated with acquired resistance to BRAFi,^5^ our results also suggest that inhibition of TMEM16A may suppress resistance to BRAFi in malignant melanoma cells. This report did not elucidate the mechanistic regulation of TMEM16A for the activation of the MEK/ERK and AKT. However, a possible regulatory scenario may be expected: the over-expressed TMEM16A in the tumor is activated by intracellular calcium induced by IP_3_/IP_3_R signal cascades and results in chloride efflux and membrane depolarization; the resultant depolarization then activates MEK/ERK and AKT kinases through versatile pathways. It would be interesting to investigate this speculation in the future.

In summary, our findings revealed that the up-regulation of TMEM16A is correlated with the constitutive activation of the MEK/ERK and AKT signaling pathways in BRAF-mutated malignant melanoma cells, resulting in a poor prognosis for patients with this tumor. Accordingly, inhibition of TMEM16A effectively suppressed the growth of A375 cells *in vitro* and *in vivo*. Moreover, TMEM16A functions cooperatively with mutated BRAF to promote malignant melanoma progression by enhancing the proliferation of the cells. We therefore propose that TMEM16A has the potential to be a therapeutic target for the treatment of BRAF-mutant malignant melanomas.

## Conflict of interests

The authors declared no potential conflicts of interest.

## References

[1] Ko J.M., Fisher D.E. (2011). A new era: melanoma genetics and therapeutics. J Pathol.

[2] Long G.V., Stroyakovskiy D., Gogas H. (2014). Combined BRAF and MEK inhibition versus BRAF inhibition alone in melanoma. N Engl J Med.

[3] Wang H., Zou L., Ma K. (2017). Cell-specific mechanisms of TMEM16A Ca^2+^-activated chloride channel in cancer. Mol Cancer.

[4] Song Y., Gao J., Guan L., Chen X., Gao J., Wang K. (2018). Inhibition of ANO1/TMEM16A induces apoptosis in human prostate carcinoma cells by activating TNF-α signaling. Cell Death Dis.

[5] Zuo Q., Liu J., Huang L. (2018). AXL/AKT axis mediated-resistance to BRAF inhibitor depends on PTEN status in melanoma. Oncogene.

