# Supplementary material for: Targeting TMEM16A/ANO1 inhibits the progression of BRAF mutant (V600E) melanoma through the MEK/ERK and AKT signaling pathways
Source: Genes Dis. 2023 Oct 27;11(6):101153. doi: 10.1016/j.gendis.2023.101153 (PMC11278794; doi:10.1016/j.gendis.2023.101153)
Supplement: Multimedia component 1 [file mmc1.docx]

**Supplementary material**

**Targeting TMEM16A/ANO1 inhibits the progression of BRAF mutant (V600E) melanoma through the MEK/ERK and AKT signaling pathways**

Meiyun Shi^#^, Jiahui Wang^#^, Xingyu Huang, Linsu Liu, Lutao Liu, Na Zhou, Xinqi Shan, Huaqun Chen*

Jiangsu Key Laboratory for Molecular and Medical Biotechnology, College of Life Sciences, Nanjing Normal University, Nanjing 210023, China.

^#^These authors contributed equally to this work.

* Correspondence: Nanjing Normal University College of Life Sciences, 1 Wenyuan Rd., Nanjing 210023, China. Telephone: 86-25-85891050. E-mail: [chenhuaqun@njnu.edu.cn](mailto:chenhuaqun@njnu.edu.cn).

**Running title: Targeting TMEM16A/ANO1 inhibits the progression of melanoma**

**Materials and Methods**

**Reagents and cells**

T16inh-A01 (Selleck, China) or CaCCinh-A01(MCE, China) was dissolved in DMSO as a stock solution (10 mM). TMEM16A antibody was purchased from Abcam (UK). p-MEK/MEK, p-ERK1/2/ERK1/2 and p-AKT/AKT antibodies were purchased from CST (USA). β-Tubulin antibody was obtained from Bioword (China).

The human melanoma cell line A375 and the control normal nevus cell line PIG1 were obtained from ATCC (kindly provided by Professor Liu Chang from Chinese Pharmaceutical University). The human prostate cancer cell line DU145 was obtained from ATCC (kindly provided by Professor Liu Ping from Nanjing Normal University). The human embryonic kidney cell line HEK293T was obtained from ATCC (USA). The cells were grown in DMEM (Gibco, USA) containing 10% fetal bovine serum (Gibco, USA) and antibiotics (100 μg/mL streptomycin and100 U/mL penicillin) (Gibco, USA), incubated at 37 ℃ with 5% CO_2_.

**MTT Assay**

The cells were inoculated at a density of 1000 cells per well (100 μL) into 96-well plates. 2 h later when the cells have adhered to the bottom of the plate, the cells were treated with different concentrations (3 μM, 10 μM, 20 μM, 30 μM, 50 μM) of the inhibitors CaCCinh-A01 or T16Ainh-A01. At 72h post treatment, MTT (5 mg/mL, 10 μL) (Sigma‒Aldrich, USA) was added to each well and incubated for another 4 h. The medium was discarded and DMSO (100μL/well) was added and incubated for 30 min. The absorbance at 490 nm was measured with a microplate reader (BioTek Synergy, USA).

**Western blot**

The samples were subjected to electrophoresis on polyacrylamide gels, and proteins were transferred onto PVDF membranes (Millipore). The membranes were then blocked with five percent (wt/vol) milk followed by probing with primary antibodies at 4℃ overnight. After incubating with HRP-labeled secondary antibodies for 1 h, the signals were visualized by BeyoECL Plus (Beyotime, China), and the images were acquired with Tanon 4500 gel imaging system (China). The intensities of the protein bands were quantified using ImageJ (USA).

**q RT-PCR**

Total RNA from cells was prepared using TRIzol reagent (Invitrogen, USA) and subjected to make cDNA using the PrimeScript RT reagent kit (TaKaRa, Japan). Quantitative real-time polymerase chain reaction (q RT-PCR) was carried out to measure the RNA expression levels of TMEM16A gene in the cells, using the SYBR Green kit (Vazyme, China). The reaction conditions were: 95℃ for 10 s, 60℃ for 30 s, 40 cycles. Amplification was performed with the StepOnePlus cycler (Applied Biosystems, USA). The RT-PCR primer sequences were listed in Table S1.

**EdU assay**

100 000 cells were plated into 24-well plates and grown overnight. Ethynyl-2ʹ-deoxyuridine (EdU) (Beyotime, China) was added into the wells and incubated for another 3 h. Subsequently, the cell nuclei were counterstained with Hoechst 33342(Beyotime, China). Images were acquired under a fluorescence microscope (Nikon, Ti-S). The EdU-positive ratio of the cells was calculated (5 fields per well).

**Preparation of the shRNA lentivirus**

For knockdown the expression of TMEM16A in A375 cells, shRNA lentiviruses were prepared. The lentiviral vector pLV3ltr-Zsgreen-puro-U6 was used to construct the TMEM16A shRNA1, shRNA2, shRNA3 and negative control shRNA plasmids. The shRNA oligos were designed and produced by Corues Biotechnology (China) based on the target sequences (Table S1). A scrambled shRNA was used as the negative control (NC). The oligos and the vector were digested with the BamHI and EcoRI enzymes, and the resultant oligos were inserted into the pLV3ltr-Zsgreen-puro-U6 vector plasmid. For lentivirus preparation, HEK293T cells were transfected using Lipofectamine 3000 (Invitrogen, USA) with the TMEM16A shRNAs or scrambled negative control shRNA and packaging plasmids. The supernatants containing viral particles were collected at 48 h and 72 h of transfection. A375 cells were transduced with the prepared lentiviruses in the presence of puromycin (2.5 µg/ml).

**Formation of xenograft tumors in nude mice**

4-week-old BALB/c nude mice (male) were obtained from GemPharmatech (China). All animals were handled and housed in accordance with the guidelines of the Animal Care Committee of Nanjing Normal University. The experiments were approved by the Experimental Committee of Nanjing Normal University (No: IACUC-1903023; Approval date: 2023-2-11). The mice were allowed to acclimate for 1 week prior to the experiment.

3 × 10^6^ A375 cells transduced with either TMEM16A shRNA3 lentiviruses or the NC lentiviruses were suspended in PBS (0.1 mL) and injected into the right flank of each mouse. Tumor growth was monitored every 2 days. From the 7th day, when tumors became visible, tumor volumes were assessed every other day by measuring two perpendicular dimensions using a Vernier caliper. The tumor size was calculated based on the formula: volume = (length × width^2^)/ 2^4^. Body weights were measured every other day. Nineteen days after inoculation, the animals were put to death by cervical dislocation; the formed tumor masses were removed, weighed and frozen in liquid nitrogen. The samples were stored at -80 °C for subsequent examination.

**Statistical Analysis**

All data were analyzed and presented as the mean ± SEM values using GraphPad Prism 6.0 software (San Diego, USA). One-way ANOVA was used to calculate p values. p < 0.05 was considered a statistically significant difference.

**Abbreviation listed in Figures**

A375, human melanoma cell line; PIG1, normal nevus cell line;DU145, human protast cancer cell line; HEK293T, human embryonic kidney cell line; CaCCinh-A01, Calcium activated chloride channel inhibitor-A01; T16Ainh-A01, TMEM16A inhibitor-A01; NC, scrambled shRNA; VMF, Vemurafenib; TPM, transcripts per million.

**Table S1 RT-PCR primers sequences and TMEM16A shRNA sequences.**

| RT-PCR primer sequence |
| --- |

Primer sequences (5’-3’)

| TMEM16A F GAGCCAAAGACATCGGAATCTG  TMEM16A R TGAAGGAGATCACGAAGGCAT |
| --- |

GAPDH F CAGGAGGCATTGCTGATGAT

GAPDH R GAAGGCTGGGGCTCATTT

| TMEM16A shRNA oligo sequences | | |
| --- | --- | --- |
| shRNA 1 GGAGTCGGGTTTGTGAAAA  shRNA2 GCTCCAGAAATCACAGAT  shRNA3 ATGCGCCACCATGGATGAA | | |
|  |  |  |


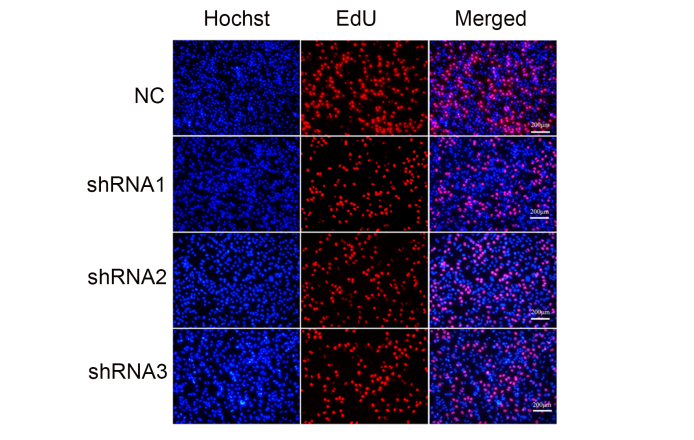


**Figure S1** EdU assay for shRNA lentiviruses transduced A375 cells. EdU positive nuclei (red) and Hoechst-stained nuclei of all the cells (blue) were visualized by fluorescence microscopy. Scale bars: 200μm.


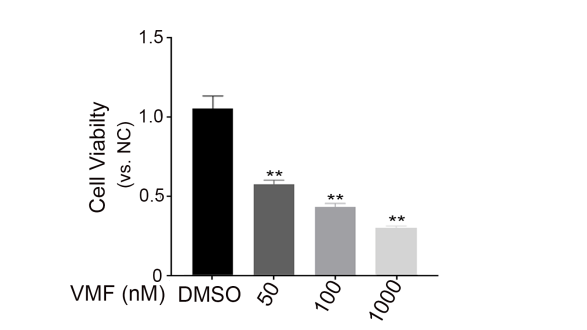


**Figure S2** MTT assay for A375 cells treated with BRAF inhibitor VMF. n = 3. ** p < 0.01.
